# Systemic Immune–Inflammatory Index and Other Inflammatory Marker Variations in Oral Squamous Cell Carcinoma Management

**DOI:** 10.3390/medicina60111840

**Published:** 2024-11-08

**Authors:** Adrian Nicoară, Ciprian Roi, Alexandra Roi, Alexandru Cătălin Motofelea, Marina Rakitovan, Flavia Zară, Mircea Riviș

**Affiliations:** 1 Department of Anesthesiology and Oral Surgery, “Victor Babes” University of Medicine and Pharmacy, Eftimie Murgu Sq. No. 2, 300041 Timisoara, Romania; nicoara.adrian@umft.ro; 2 Department of Anesthesiology and Oral Surgery, Multidisciplinary Center for Research, Evaluation, Diagnosis and Therapies in Oral Medicine, “Victor Babes” University of Medicine and Pharmacy, Eftimie Murgu Sq. No. 2, 300041 Timisoara, Romania; rivis.mircea@umft.ro; 3 Department of Oral Pathology, Multidisciplinary Center for Research, Evaluation, Diagnosis and Therapies in Oral Medicine, “Victor Babes” University of Medicine and Pharmacy, Eftimie Murgu Sq. No. 2, 300041 Timisoara, Romania; alexandra.moga@umft.ro; 4 Department of Internal Medicine, Faculty of Medicine, “Victor Babes” University of Medicine and Pharmacy, 300041 Timisoara, Romania; alexandru.motofelea@umft.ro; 5 Department of Microscopic Morphology, Faculty of Medicine, “Victor Babes” University of Medicine and Pharmacy, 300041 Timisoara, Romania; marina.rakitovan@umft.ro (M.R.); flavia.zara@umft.ro (F.Z.)

**Keywords:** inflammatory markers, systemic immune–inflammation index, neutrophil lymphocyte ratio, oral squamous cell carcinoma, TNM

## Abstract

*Background and Objectives*: With the greatest rate of morbidity and death, OSCC is one of the world’s most critical public health problems. Being a complex pathology, the management process that includes diagnostic, surgical, and adjuvant treatments must as well take into account the involvement of the immune system. This study aims to evaluate various biomarkers such as neutrophils, lymphocytes, platelets, SII, and NLR in the different stages of OSCC treatment and in correlation with TNM stages, in order to observe the inflammatory response of the host. *Materials and Methods*: A total of 154 patients diagnosed with OSCC were included in the present retrospective study. Routine blood samples were collected from all patients both before and after surgery. Using the detected values of platelets, neutrophils, and lymphocyte count, the systemic immune–inflammation index (SII) and neutrophil-to-lymphocyte ratio (NLR) were calculated. Based on the oncologist’s recommendation, 46 patients underwent adjuvant radiotherapy as part of their oncologic treatment plan. For these patients, additional blood samples were collected before the first and after the last radiotherapy session for determining the values of platelets, neutrophils, and lymphocyte count, and SII and NLR calculation. *Results*: Prior to the first radiotherapy session, neutrophils decreased slightly to 4.35, lymphocytes increased to 2.23, and platelets rose to 258.62. The SII and NLR were 641.02 and 2.19, respectively. Following the last radiotherapy session, neutrophils increased substantially to 10.30, while lymphocytes decreased to 1.21. Platelets showed a slight reduction to 227.08. Notably, the SII rose dramatically to 3084.19, and the NLR increased significantly to 15.49, suggesting an important immune and inflammatory response of the host. *Conclusions*: The host’s immunological and inflammatory responses are impacted by both surgery and adjuvant radiation administered following surgery. The parameters assessed—neutrophils, lymphocytes, platelets, SII, and NLR—qualify as significant variables that need to be monitored before, during, and following OSCC therapy. This study’s findings validated significant changes in immunological and inflammatory markers in the management of OSCC.

## 1. Introduction

With a concerning increase during the last decades, cancer is a disease with a high morbidity and mortality rate, posing as one of the most concerning public health issues worldwide [1,2,3,4,5]. The squamous cell carcinoma of the head and neck (HNSCC) is the seventh most frequently diagnosed tumor worldwide, responsible for approximately 4.5% of all cancers and 4.6% of cancer-related deaths globally, with ever-rising incidence and mortality rates [5,6,7,8].

Among head and neck cancers (HNCs), oral squamous cell carcinoma (OSCC) is the most prevalent malignant tumor, ranking 16th place worldwide. Its aggressive nature, characterized by a high recurrence and metastatic potential, makes it one of the most challenging malignant tumors to treat [1,2,5,9,10,11].

Even though there have been improvements in the multidisciplinary collaboration for a proper treatment, the therapeutic protocol for OSCC includes surgery, radiotherapy, chemotherapy, molecularly targeted drugs, and immune checkpoint inhibitors. Currently, surgical resection is still considered an elective therapy [5,9,12,13].

Due to continuous advancements in the diagnosis steps and protocols, as well as treatment technologies, the survival rate and prognosis for OSCC have significantly improved [8,9,14]. Still, there remains the need to explore new tools that can estimate more accurately the therapeutic approaches and prognosis in order to improve the patients’ living standards [8,9,14,15,16]. For this purpose, there is the need to develop and analyze biomarkers that can evaluate and predict the prognosis and therapeutic response, to implement guided therapies meant to achieve more effective treatments [5,14,16].

The inflammatory process is closely associated with all stages of the development and malignant progression of cancer, as well as with the efficacy of anti-cancer therapies [17]. Several inflammatory biomarkers have been reported as prognostic factors in various carcinomas [14,18].

Using a series of inflammatory biomarkers is a cost-effective and easily identifiable prognostic indicator at the time of diagnosis, ensuring its accessibility and affordability for broad applications in clinical use [5,19].

Using peripheral blood samples and various serum systemic inflammatory response markers can be efficient for numerous malignancies. For example, the neutrophil-to-lymphocyte ratio (NLR) has been found as a cost-effective marker correlated with the survival rate in oral cancer, while the dynamic monitoring of NLR can be used as a tool in predicting OSCC prognosis [14,16,20,21].

The systemic immune–inflammation index (SII) is another relatively novel inflammatory biomarker, used in a multitude of pathologies to assess systemic inflammation and it is suggested to properly indicate the balance between host inflammation and the immune status [14,22,23].

The SII is easily calculated, from routine blood tests, as the ratio between peripheral neutrophils × platelets/lymphocytes. The SII has been analyzed as a prognostic tool in a wide range of pathologies, like in hepatocellular, gastric, colorectal, small cell lung cancer, thyroid cancer, and type 2 diabetic retinopathy, as well as a predictive marker for life-threatening cervicofacial infections, cardiovascular disease, and as a mortality cardiovascular predictor [14,19,23,24,25,26,27,28].

While in recent years many researchers have underlined the SII’s potential in forecasting disease severity and monitoring the efficacy of treatments, related to multiple pathologies, there are still few studies that have focused on the importance of evaluating the SII in oral cancer [14,19,25].

The present study aims to examine the impact of oncological treatment, a combination of surgery and radiotherapy, in patients with OSCC on the NLR, SII, and individual levels of blood count fractions (platelets, neutrophils, and lymphocyte), and identify potential correlations between these biomarkers, the severity of the cases, and the immune and inflammatory response of the host.

## 2. Materials and Methods

The present study is a retrospective one, based on diagnosed cases of oral squamous cell carcinoma patients admitted in the Oro-Maxillo-Facial Surgery Clinic of the Emergency City Hospital, Timișoara, between January 2012 and December 2022. The data were collected from the medical charts and were organized by age, gender, diagnosis, treatment, bloodwork values, and clinical and paraclinical examination outcomes.

The present study was approved by the Ethics Committee of University of Medicine and Pharmacy “Victor Babeș” Timișoara (No. 48/10 September 2024), and an informed consent form was signed by each patient, following the guidelines of the Declaration of Helsinki.

### 2.1. Patient Recruitment

Inclusion criteria:

- Age range between 18 and 90 years;
- Both sexes;
- Diagnosis of OSCC;
- Hospitalized patients in the Oro-Maxillo-Facial Surgery Clinic;
- Patients with a clear indication for surgical treatment;
- Patients who received surgical and postoperative radiotherapy treatment.

Exclusion criteria:

- Age < 18 years;
- Incomplete medical charts;
- Pregnancy;
- Absence of postoperative radiotherapy.

### 2.2. Blood Samples

In accordance with Oro-Maxillo-Facial Surgery Clinic protocol, patients were admitted and examined. For the first group of patients (*n* = 154), venous blood from the antecubital vein was drawn preoperatively and postoperatively. In the second group (*n* = 46), venous blood was collected at first preoperatively, the second time, postoperatively, right before the first radiotherapy session, and again, for the third time, following the final radiotherapy session.

Routine blood tests were conducted immediately after collecting the samples to determine biomarkers. For the systemic immune–inflammation index (SII) calculation, the used reference values were as follows: neutrophil counts (2.04–7.60 × 10^3^/μL), platelet counts (150–410 × 10^3^/μL), and lymphocyte counts (1.0–3.0 × 10^3^/μL). The SII formula applied was SII = (neutrophil × platelet)/lymphocyte counts, with the results expressed as ×10^3^/μL.

For calculating the neutrophil-to-lymphocyte ratio (NLR), the used reference ranges were as follows: neutrophil counts (2.04–7.60 × 10^3^/μL) and lymphocyte counts (1.0–3.0 × 10^3^/μL). The NLR was determined by dividing the neutrophil count by the lymphocyte count. A normal NLR range falls between 1 and 2, with values above 3.0 or below 0.7 in adults considered abnormal [20].

Upon admission, blood test data were recorded, including neutrophil, lymphocyte, and platelet counts (150.0–410.0 × 10^3^/μL).

### 2.3. Patient Management

The entire lot of 154 patients were treated surgically at the Oro-Maxillo-Facial Clinic in Timișoara, after establishing the diagnosis of OSCC and the indication for surgery. Routine blood samples were taken from all patients. Using the detected values of platelets, neutrophils, and lymphocyte count, both before and after surgery, the systemic immune–inflammation index (SII) and neutrophil-to-lymphocyte ratio (NLR) were calculated. After the patients’ hospital discharge, based on the histopathological result, all patients attended an oncological examination to determine the necessity of further treatment, such as radiotherapy.

Based on the oncologist’s recommendation, 46 patients underwent adjuvant radiotherapy as part of their oncologic treatment plan. For these patients, additional blood samples were collected, and SII and NLR were calculated, after the completion of radiotherapy. 

This protocol enabled a comprehensive analysis of the patients’ immune and inflammatory responses at various treatment stages.

### 2.4. Radiotherapy

The definition of target volumes and organs at risk is standardized by ICRU norms, using target volumes for primary tumor (T) and regional lymph nodes (N), as follows [29]:

- Gross tumor volume (GTV T and GTV N).
- Clinical target volume (CTV T and CTV N).
- Planning target volume (PTV T and PTV N).

In the group of patients included in this study that benefitted from postsurgical adjuvant radiotherapy for OSCC in the Radiotherapy Clinic of the Emergency City Hospital, Timișoara, the volumetric modulated arc therapy (VMAT) technique was used for defining the CTV T, to facilitate the three-dimensional (3D) conformational postoperative adjuvant external radiotherapy.

The X-ray emission source used was the CLINAC linear accelerator, adapting the radiotherapy to the patient’s needs. The system uses conformational 3D scans of the body, further modeling the irradiation field, according to the irregular shape and the targeted tumor volume, with the help of the multilayer collimation system (MLC), directing the radiation beam from various fixpoints. The dosage prescriptions used were 66 Gy/33 fx for high-risk areas, 60 Gy/30 fx for intermediate, and 50 Gy/25 fx for low-risk areas. The treatment was initiated within 6 weeks postoperatively.

### 2.5. Statistical Analysis

Continuous variables were assessed for normality using the Shapiro–Wilk test. Normally distributed data were presented as means ± standard deviations (SDs), while non-normally/abnormally distributed data were summarized using medians with interquartile ranges (IQRs; 25th to 75th percentiles). Categorical variables were described as counts and percentages. Differences between groups for normally distributed continuous variables were assessed using Welch’s *t*-test for comparisons between two groups and one-way ANOVA for multiple groups, incorporating post-hoc tests (e.g., Tukey’s HSD) to pinpoint specific group differences. For non-parametric continuous data, the Mann–Whitney U and Kruskal–Wallis tests were applied for two and multiple groups, respectively, with subsequent Dunn’s post-hoc analyses as necessary. Categorical data comparisons were conducted using Chi-square tests or Fisher’s exact tests when expected cell counts were below five. Sample size calculations were conducted a priori to achieve a confidence level of 95% and a statistical power of 80% based on anticipated effect sizes and variance estimates derived from preliminary data. All statistical analyses were performed in R (version 3.6.3), leveraging the capabilities of several comprehensive packages within the Tidyverse for data manipulation and visualization. We used specialized packages, including MCGV, Stringdist, Janitor, and Hmisc, for various data processing needs. Additionally, we utilized ggplot2 to create graphical boxplots and violin plots.

## 3. Results

### 3.1. Preoperative Statistical Analysis

A total of 154 patients were analyzed in the first part of this study. The patient distribution among the five groups was realized based on the T classification (T1, T2, T3, T4, and T4a), as detailed in Table 1.

The mean age showed no significant statistical differences between the groups (*p* = 0.719). The mean age across all groups was 62.3 years (SD = 10.1), with the T1 group having a slightly higher average age of 63.7 years (SD = 9.6) and the T4 group presenting with a lower mean age of 60.3 years (SD = 16.8).

Gender distribution was consistent across groups, with no statistically significant differences (*p* = 0.9801). The majority of the patients were male (72%), with the male distribution ranging from 67.3% in the T1 group to 100% in the T4 group.

The analysis of tumor–node–metastasis (TNM) node involvement revealed statistically significant differences between groups (*p* < 0.0011). Most of the patients had no lymph node involvement (N0 = 80.6%). However, higher TNM stages (N2, N3) were observed more frequently in the T3 and T4 groups.

Preoperative neutrophil counts were consistent across the groups (*p* = 0.3232), with a mean value of 5.2 (SD = 2.0). Similarly, lymphocyte counts showed no significant differences (*p* = 0.8262), with an average of 2.0 (SD = 1.2).

Platelet counts approached statistical significance between groups (*p* = 0.0832), with the mean platelet count being 243.0 (SD = 66.9). The T1 group showed a higher platelet count (261.7), while the T3 group had the lowest mean (219.1).

The SII showed no statistically significant differences between the groups (*p* = 0.4352), with the overall mean being 756.0 (SD = 548.4).

A total of 154 patients were analyzed across multiple histopathological pleomorphic differentiation grades (G1, G1/G2, G2, and G3). The most common differentiation grade was G2, observed in 102 patients (66.7%). G3 was the least common grade, with only 7 patients (4.6%). No statistically significant differences in tumor grade were observed regarding the preoperative neutrophil count, lymphocyte count, or platelet count (*p* > 0.05 for all variables).

Similarly, the platelet count did not show statistically significant variations (*p* = 0.8521). The overall mean platelet count was 243.0 (SD = 66.9), with the N3 group showing the highest value (305.0) and the N2 group having the lowest (204.0).

The SII was not significantly different between groups (*p* = 0.9741), with an overall mean of 756.0 (SD = 548.4). Patients in the N2c group had the highest mean SII (1009.1), while patients in the N3b group had the lowest (477.9).

The NLR also showed no significant differences (*p* = 0.9981), with a mean of 3.0 (SD = 1.8). The N3b group had the lowest NLR (1.8), while the N2c group had the highest (3.7).

Statistically significant differences were observed in tumor size classification (*p* < 0.0012). The majority of patients were classified as T2 (47.7%) and T1 (36.1%). Patients in the more advanced N-stages (N2, N3) tended to have larger tumors (T3, T4); within the N2b group, T3 tumors had the higher proportion (23.1%).

No statistically significant differences were found in the distribution of non-keratinized squamous cell carcinoma (*p* = 0.8422). The majority of patients (77.4%) did not present with this form, although it was slightly more common in the N1 group (33.3%).

Similarly, keratinized squamous cell carcinoma did not differ significantly between groups (*p* = 0.8422). Most patients (77.4%) had keratinized squamous cell carcinoma, with the highest occurrence in patients with more advanced lymph node involvement (100% in N2b and N3).

The tumor grade did not show statistically significant differences across the groups (*p* = 0.1512). The majority of patients were classified as G2 (66.7%). Patients with more advanced lymph node stages (N2, N3) exhibited a higher frequency of G2 or G3 tumors.

### 3.2. After Surgical OSCC Extirpation Statistical Analysis

Neutrophil counts increased post treatment, but neither the ANOVA (*p* = 0.4290) nor the Kruskal–Wallis test (*p* = 0.0867) indicated significant differences, suggesting the variation observed is not statistically meaningful.

Lymphocyte counts showed a significant decrease from the preoperative period (mean = 2.03) to the post-last session of radiotherapy (Mean = 1.17), with both the ANOVA and Kruskal–Wallis tests showing highly significant results (*p* < 0.0001). This indicates a notable impact on the lymphocyte levels, potentially reflecting an immunosuppressive effect.

Platelet counts fluctuated slightly over time, with no significant differences according to the ANOVA (*p* = 0.3594) or Kruskal–Wallis test (*p* = 0.2554). This suggests that platelet levels remained relatively stable across the sessions.

The SII showed a substantial increase post-last session, with the Kruskal–Wallis test (*p* = 0.0019) identifying significant changes despite the ANOVA result being non-significant (*p* = 0.2266). The increase in SII may indicate a pronounced inflammatory response following treatment.

The NLR rose significantly post treatment, with the Kruskal–Wallis test showing a highly significant change (*p* < 0.0001), while the ANOVA showed no significant changes (*p* = 0.2419). The marked increase in NLR suggests an acute inflammatory response after the last session of radiotherapy.

### 3.3. After Radiotherapy Treatment Statistical Analysis

The second part of this study includes only patients from the first lot that have been diagnosed with OSCC and had surgical and radiotherapeutic treatment: 46 patients—12 females (26.1%) and 34 males (73.9%). A total of 108 patients were excluded due to the absence of postoperative radiotherapy. The mean age of the subjects included in this analysis was 63.33 years (SD = 9.63), the youngest patient being 44 years old and the oldest 88.

The distribution of patients regarding the TNM clinical staging of the OSCC at the time of hospital admission was as follows: T1 (tumor 2 cm or less in greatest dimension) in 15 patients (32.6%), T2 (tumor more than 2 cm but not more than 4 cm in greatest dimension) in 25 patients (54.3%), T3 (tumor more than 4 cm in greatest dimension) in 4 patients (8.7%), and T4a (moderately advanced local disease) in 2 (4.3%) patients.

The distribution of lymph node metastases in the case of the patients included in this study was as follows: N0 (no regional lymph node metastasis) in 37 (80.4%) patients, N1 (metastasis in a single ipsilateral lymph node, 3 cm or less in greatest dimension) in 2 (4.3%) patients, N2a (metastasis in single ipsilateral lymph node more than 3 cm but not more than 6 cm in greatest dimension) in 1 (2.2%) patient, and N2b (metastasis in multiple ipsilateral lymph nodes, none more than 6 cm in greatest dimension) in 6 (13%) patients, this representing the largest group with nodal metastasis.

The distribution of patients regarding the histopathological form of the OSCC was as follows: keratinized oral squamous cell carcinoma in 28 cases (60.9%) and non-keratinized squamous cell carcinoma in 18 cases (39.1%). The distribution of patients regarding the histopathological tumor differentiation/from a descriptive nuclear pleomorphism point of view was as follows: G1 in 7 (15.9%) patients, G1/G2 in 1 (2.3%) patient, G2 in 33 (75%) patients, G2/G3 in 2 (4.6%) patients, and G3 in 1 (2.3%) patient.

Preoperative blood parameters showed mean neutrophil levels of 5.01 (SD = 1.89), lymphocytes at 2.05 (SD = 0.68), and platelets at 233.02 (SD = 58.65). The SII was 668.69 (SD = 483.28) and the NLR was 2.82 (SD = 1.75), as shown in Table 2.

Prior to the first radiotherapy session, neutrophils decreased slightly to 4.35 (SD = 2.07), lymphocytes increased to 2.23 (SD = 0.68), and platelet values increased to 258.62 (SD = 110.67). The SII and NLR were 641.02 (SD = 876.85) and 2.19 (SD = 1.50), respectively.

**Figure 1:**
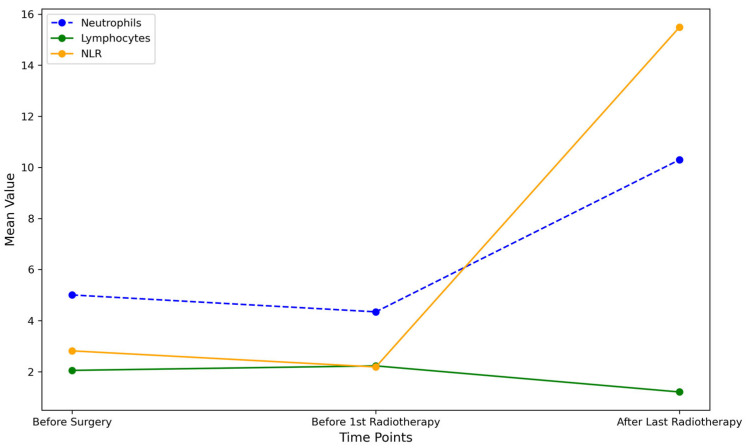
Overlay of mean trends for neutrophils, lymphocytes, and NLR across treatment stages.

Following the last radiotherapy session, neutrophils increased substantially to 10.30 (SD = 40.27), while lymphocytes decreased to 1.21 (SD = 0.71). Platelets showed a slight reduction to 227.08 (SD = 98.20). Notably, the SII rose dramatically to 3084.19 (SD = 13,001.48), and NLR increased significantly to 15.49 (SD = 71.06) (Figure 1).

**Figure 2:**
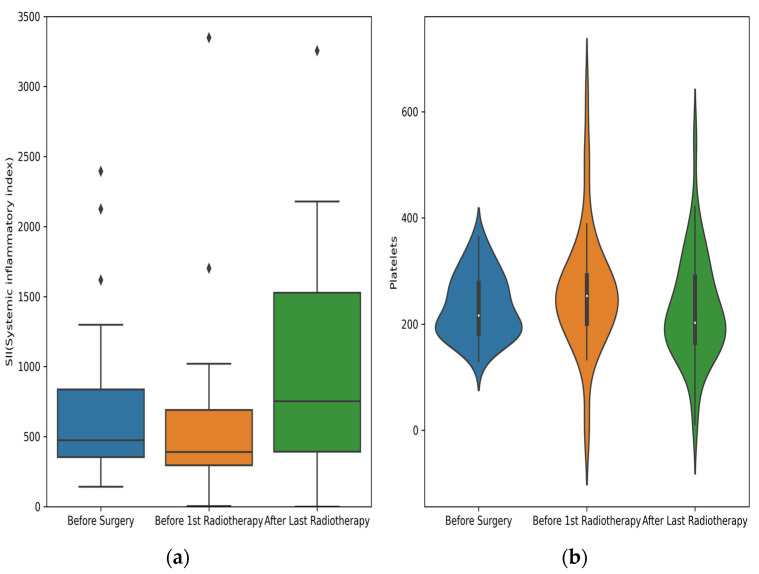
Boxplot for SII across treatment stages: pre surgery, pre radiotherapy, and post radiotherapy (**a**); violin plot for platelets across treatment stages: pre surgery, pre radiotherapy, and post radiotherapy (**b**).

These data suggest notable changes in immune and inflammatory markers post radiotherapy, especially in neutrophil counts, SII, and NLR, which may reflect an acute inflammatory response. The patient population also displayed a predominance of T2 tumors with mostly moderate differentiation (G2) (Figure 2).

## 4. Discussion

This study aims to evaluate the impact of oncological treatments, specifically combining surgery and postoperative adjuvant radiotherapy, on the immune and inflammation response of the host. We analyzed the individual and combined changes in various serum markers, with special attention to the variation of the NLR and SII in the management of OSCC patients. Additionally, the potential correlation between these biomarkers and the severity of the cases was explored.

Even though, due to continuous advancements in the diagnosis, the examination protocols, and treatment technologies, the survival rate and prognosis of OSCC has significantly improved during the last years, surgical resection is still an elective therapy [8,9,14]. The therapeutic protocol for OSCC includes, besides surgery, radiotherapy, chemotherapy, molecularly targeted drugs, and immune checkpoint inhibitors [14,15].

To this day, the oral and maxillofacial surgeons encounter difficulties in evaluating the prognosis of OSCC patients because the main framework for treatment planning and survival outcomes is still the TNM classification [5,14,25,30,31,32,33]. However, this widely used clinicopathological staging system has certain limitations [5,25]. Even among patients with the same TNM stage, the postsurgical evolution of the disease can vary significantly, since the progression of the case is altered by a multiple factors. Numerous studies have demonstrated the complex involvement and constant presence of inflammatory reactions in all stages of cancer development and also in the efficacy of oncological therapies [17,32]. Inflammatory reactions were found to have a decisive role in shaping tumoral development at different stages of cancer, ranging from the tumoral initiation, progression of cancer, tissue infiltration, and tumoral metastasis [1,5,14,26,27]. Detailed research noted a significant link between systemic inflammatory related peripheral cells (neutrophils, lymphocytes and platelets) derived from the peripheral blood in case of the oncological patients [25,26]. Therefore, given the strong relationship between inflammation and malignancy, targeting inflammation parameters is an important tool of predicting oncological outcome and an important way of optimizing oncological treatment [17,27,30].

Platelets are capable of mediating tumor cell growth and survival at distant sites [27]. This potential to facilitate progression and metastatic spread of the tumor has been linked to their ability to promote adhesion, due to their capacity to create a mechanical support, stimulate the angiogenesis process and vessel permeability, promote distant metastasis, thereby forming new metastatic niches [5,8,14,27,33]. Also, platelets show a capacity to prevent cell death by forming a physical shield around cancer cells, protecting tumor cells from immune destruction and reducing the cytotoxic activity of natural killer cells [5,14,26,33]. So, elevated platelet levels are positively associated with poor survival in cancer [8,33].

In this study, the preoperative platelet count showed a mean value of 233.02 (SD = 58.65), with an increased postsurgical value of 258.62 (SD = 110.67) and an additional slight reduction to 227.08 (SD = 98.20) after the completion of radiotherapy. Furthermore, studies have established that platelets have a contribution to the induction of tumor-associated neutrophils [27].

Additionally, studies indicate that an increased number of neutrophils are associated with the invasion in the tumor microenvironment, causing the host to lose its ability to effectively target tumor cells, indirectly facilitating tumor progression and therefore, leading to an unfavorable prognosis in cancer patients [5,8,14,15,26,30,31]. Neutrophils are induced by vascular endothelial growth factors expressed by the cancer cells, while they are able to secrete cytokines and chemokines, which are highly associated with carcinogenesis and create a favorable habitat for tumor growth [14,25,30,33].

In this study, preoperative blood parameters showed mean neutrophil levels of 5.01 (SD = 1.89), with a slight decrease to 4.35 (SD = 2.07) before the first radiotherapy session, and a substantial increase after completing radiotherapy, ranging up to 10.30 (SD = 40.27). Neutrophils also have a suppressive action on the activity of lymphocytes [14].

Lymphocytes are fundamental cellular components of the immune system that play an important role in the adaptive immune response by maintaining body equilibrium, balancing the body’s defense and noxious agents involved in many diseases, including malignant tumors [5,14,27,30]. Although lymphocytes may not always be active in cancers due to immune escape or tolerance, T cell activation allows tumor-infiltrating T lymphocytes to induce apoptosis in tumor cells, leading to their death in response to radio or/and chemotherapy [14,32]. The presentation of tumor-associated antigens to lymphocytes leads to the conclusion that higher lymphocyte levels represent a key beneficial factor in adjuvant therapies, being associated with the prevention of tumoral recurrence and is positively associated with a better prognosis in cancer patients [8,14,32,33].

In the present study, preoperative blood parameters showed lymphocyte levels with an overall mean of 2.05 (SD = 0.68), the lowest values being in the N3B group (1.3) and a slight increase to 2.23 (SD = 0.68) postoperatively. Lymphocyte counts showed a significant decrease from the preoperative period to the levels after the completion of radiotherapy (mean = 1.17), with both the ANOVA and Kruskal–Wallis tests indicating highly significant results (*p* < 0.0001). This suggests a notable impact on lymphocyte levels, potentially reflecting an immunosuppressive effect.

Utilizing various combinations of different inflammation-related markers as prognostic indicators, these will provide more stable and reliable results than using an individual blood fraction [5,22,30,33].

Alongside platelets, neutrophils, and lymphocytes, the present study also aimed to evaluate the impact of surgery combined with radiotherapy on the NLR and SII in the treatment of OSCC patients.

The NLR has been extensively studied as a prognostic marker in head and neck malignancies, being considered a better indicator of the balance between tumor growth environment and immunity compared to individual blood count fractions, with significant prognostic value in patients with HNCs [8,14,34]. Numerous studies have shown that a high preoperative NLR is significantly associated with an increased risk of death and low survival rate in primary OSCC [30].

Also in oropharyngeal cancer, a high NLR in patients is associated with a worse 5-year disease-free survival, while a high NLR at 3 months after completion of radiotherapy was also associated with a low survival [34,35]. Moreover, pre-treatment NLR has been correlated with cancer-related mortality, and the NLR is now considered a reliable prognostic marker not only in malignancies but also in various benign conditions [24,34,35,36].

In the present study, the NLR showed preoperative mean levels of 2.93 (SD = 1.85), with a slight decrease to 2.15 (SD = 1.47) postoperatively, before the first radiotherapy session, and a significant increase after the completion of radiotherapy to 16.49 (SD = 73.65). The NLR had risen significantly post treatment, with the Kruskal–Wallis test showing a highly significant change (*p* < 0.0001), while the ANOVA test did not indicate any significance (*p* = 0.2419). The marked increase in NLR suggests an acute inflammatory response after the last session.

Many authors have suggested that the SII is superior to other existing systemic inflammation- and immune-based prognostic indexes such as the PLR, LMR or NLR. They even suggest that it is more sensitive and specific in prognostic prediction and is a more objective, comprehensive, and reliable marker that reflects better the balance between host inflammation and immune status, and has proven to be more valuable in predicting overall survival across various malignant tumors [8,14,15,25,26,30,32,33,37].

The SII is validated to be an improved index that can indicate more accurately the balance between the host inflammation and the immune status, while in oncological patients, it provides an objective measure of variation in the balance between the body’s anti-tumor immune response and systemic inflammation linked to cancer [8,14,15,23].

While there are studies that have reported some inflammation-based prognostic scores (like the NLR, PLR, or LMR) as being useful in oral cancer, several studies have focused on the SII’s importance as a prognostic factor in head and neck or oral malignant tumors [5,8,14,15,25,30]. Lu Z. et al. reported that pre-treatment SII has great potential in improving the accuracy of clinical prognosis, being a useful prognostic factor for overall survival and even tumor-free survival in SCC of the tongue and in patients undergoing primary site surgery and cervical dissection [25]. Both Diao et al. and Zhan et al. concluded that elevated SIIs are significantly associated with poor outcomes, poor overall survival, and disease-free survival, and suggested that the SII could serve as a non-invasive, low-cost and powerful prognostic predictor, and is a potentially valuable biomarker for the clinical management and prognostic prediction of OSCC patients [5,30].

Diao et al. found that the SII and NLR are significantly associated with overall and disease-free survival and served as independent prognostic predictors with satisfactory sensitivity and specificity for OSCC [30].

In this study, the SII showed preoperative mean levels of 703.93 (SD = 505.66), with a slight postoperative decrease to 645.30 (SD = 915.50) before the first radiotherapy session. In the present study group, the SII was not significantly different between groups (*p* = 0.9741). Patients in the N2c group had the highest mean SII (1009.1), while patients in the N3b group had the lowest (477.9).

The study findings are, in part, consistent with the findings of Wang et al., who identified that an elevated pre-treatment SII is correlated with a more advanced tumor and nodal status, having a lower survival outcome in cases of HNC [8].

After the completion of radiotherapy, the SII rose dramatically to 3293.11 (SD = 13,468.30). The SII showed a substantial increase post-last session, with the Kruskal–Wallis test (*p* = 0.0019) identifying significant changes, despite the ANOVA result being non-significant (*p* = 0.2266). The increase in the SII may indicate a pronounced inflammatory response following radiotherapy treatment.

Even if the association between the SII and cancer prognosis is controversial, and in particular, in OSCC, a high SII frequently has been correlated with advanced clinicopathological characteristics, representing a reliable prognostic factor for long-term survival and disease free-survival in numerous malignant tumors [5,8,14,15,25,27,30,32,33], recent findings have suggested the association of SII with a poor prognosis in cancers, being a reliable indicator of circulating tumor cell counts, and correlated with a greater metastatic potential [15,27].

Zhang et al. concluded that the SII has a stable role when predicting prognosis, with elevated SII levels clearly associated with locally advanced tumors (TNM–T3/T4) and poor tumor differentiation. Moreover, the SII was a significant predictor for the short- and long-term survival of OSCC [5].

This study’s data suggest notable changes in immune and inflammatory markers post radiotherapy, especially in neutrophil counts, SII, and NLR, which may reflect an acute inflammatory response. The patient population also displayed a predominance of T2 tumors with mostly moderate differentiation (G2).

Preoperative increased levels of neutrophils and platelets, combined with a low level of lymphocytes and an elevated SII, usually indicate an imbalance of the inflammatory response, with a stronger pro-tumoral inflammatory activity and a weaker anti-cancer immunological response in patients [8,14,25,26]. These are significantly correlated with aggressive features of cancer, like larger tumor size, poor differentiation, local tumor invasion, metastasis, and a worse prognosis, with elevated SII levels that significantly negatively are associated with the overall survival [8,14,25,26,30,32,38].

Radiotherapy is a major adjuvant method of treatment for OSCC that can trigger an immune-mediated tumor response and remodel the inflammatory microenvironment [14,32]. Additionally, increasing evidence suggests that the inflammatory tumor microenvironment plays a crucial role in determining the effectiveness of conventional treatments such as radiotherapy [17]. Research cited by Wang et al. suggests that radiotherapy can recruit inflammatory cells into the tumor microenvironment, triggering an immune response that either supports cancer cell survival or promotes their destruction. Additionally, radiotherapy is often utilized as a palliative treatment option for OSCC, with numerous studies reporting its efficacy in alleviating symptoms and improving the quality of life [32,39].

Several studies have examined the utility and superiority of the SII to other indexes and its different dynamic change patterns as a prognostic factor in tumors treated by radiotherapy or a combination of surgery and radiotherapy, and the impact of radiotherapy on SII levels in different cancers [14,32,33,38,40].

Balázs et al. identified that radiotherapy intensified the immune-suppressive characteristics, which lasted for at least a month post treatment. Understanding the impact of radiotherapy on the systemic anti-tumor immune response can enhance treatment strategies by integrating immunotherapy with radiotherapy [40].

Therefore, it is crucial to find novel potential molecular targets for the treatment of OSCC. The iron-dependent buildup of reactive oxygen species and the resulting oxidative damage to lipid membranes produce ferroptosis, a controlled cell death. An increasing body of research over the past five years has shown that OSCC is susceptible to ferroptosis induction and that ferroptosis inducers have a significant anti-tumor impact in OSCC, especially in cases where the tumor shows little response to standard treatments like chemotherapy and radiation [41].

In conclusion, early identification of reliable prognostic markers can significantly help clinical decision-making, ultimately improving patient survival and quality of life [33]. A deeper understanding of how dysregulated immune–inflammation reactions correlate with tumor progression can lead to the development of innovative strategies for combating cancer, thus improving the effectiveness of the multimodal oncological treatment [17].

However, as noted by Staniewska et al., pre-treatment blood parameters, despite their proven benefits, are infrequently utilized in prognostic evaluations [15]. Addressing this shortfall could aid in determining the most effective therapeutic strategies, leading to better outcomes for patients [32]. Given the strong relationship between surgery, radiotherapy and immune status, we proposed that the SII might correlate with the effectiveness of OSCC treatment.

As a biomarker represented by the systemic immune–inflammation, the SII and its fluctuations could provide prognostic insights for patients with OSCC undergoing ablative surgery and adjuvant radiotherapy, suggesting its potential utility in future prognostic frameworks. Moreover, the present study findings indicate that monitoring pre-treatment SII could facilitate early identification of advanced disease characteristics and tumor progression in OSCC patients.

The SII is easily measured through blood samples, making it a practical tool for daily clinical practice in personalized treatment planning for OSCC. This study demonstrates that elevated NLR and SII levels are associated with aggressive clinicopathological features, including advanced T classification and nodal metastasis. This study’s findings align with authors that have underlined the SII’s supporting role as a prognostic biomarker in HNC [8]. Since SII is a dynamic score that can vary with cancer progression and treatment interventions, regular monitoring during therapy may yield valuable insights into the inflammatory and immune status of patients and their clinical responses to therapy [30].

Incorporating the SII into prognostic assessments could enhance our understanding of patient outcomes, risks of disease progression, and therapeutic responses in clinical settings. While evaluating pre-treatment status, the SII holds promise for establishing treatment strategies in patients with OSCC [30]. More research is needed to clarify its prognostic predictive values across various treatment approaches, including combinations of surgery with radiotherapy.

Other marker variations used in prognostic or recurrence roles for OSSC are represented by the platelet–lymphocyte ratio (PLR), systemic inflammation response index (SIRI), and prognostic nutritional index (PNI) [42]. Preoperative SIRI may be a valuable tool for the prediction of the survival of OSCC patients. Patients with a higher SIRI had a significantly increased risk of mortality compared with those with a low SIRI [43]. The PLR is a readily available biomarker that will improve prognostication and risk stratification in OSCC. The PNI is a valuable independent tumor recurrence prediction index in patients with advanced OSCC. Meanwhile, the combination of preoperative SII and PNI is also valuable on OSCC recurrence and prognosis prediction [44]. 

Regarding the limitations of this study, it was a single-center study with a relatively small number of OSCC patients who underwent surgery and adjuvant radiotherapy. Nevertheless, the present study contributes to the growing body of knowledge in this field and has the potential to expand research efforts in the oncological treatment of OSCC. It could also serve as a foundation for a larger multicentric study.

Acute inflammation is also induced by anti-cancer therapies, and it contributes to the destruction of the cancer cells by inducing an anti-tumor immune response. Thus, the chronic inflammation found in post-oncological therapy leads to therapeutic resistance and even cancer progression.

## 5. Conclusions

It is critical to evaluate how oncological treatments—especially the combination of surgery and adjuvant radiation given after surgery—affect the host’s inflammatory and immunological responses. In summary, the study findings validated significant alterations in immunological and inflammatory indicators in the treatment of OSCC, and the parameters assessed—neutrophils, lymphocytes, platelets, SII, and NLR—qualify as crucial variables that need to be tracked prior to, during, and following OSCC therapy.

Owing to their outstanding qualities of affordability, simplicity, repeatability, and ease of computation, these variables hold tremendous promise as reliable and promising parameters for establishing OSCC management.

The use of these biomarkers might assist in defining therapy targets, evaluating treatment methods, and measuring therapeutic responses by providing more accurate prognostic insights, thereby enhancing patients’ quality of life.

## Figures and Tables

**Table 1:** The patient preoperative distribution statistical analysis.

	T1 (N = 55)	T2 (N = 74)	T3 (N = 15)	T4 (N = 3)	T4a (N = 7)	Total (N = 154)	*p*-Value
Age							0.719
Mean (SD)	63.7 (9.6)	61.3 (10.7)	63.3 (8.3)	60.3 (16.8)	61.1 (9.2)	62.3 (10.1)	
Range	43.0–88.0	36.0–87.0	51.0–82.0	42.0–75.0	51.0–71.0	36.0–88.0	
SEX							0.980
F	18 (32.7%)	21 (28.4%)	3 (20.0%)	0 (0.0%)	1 (14.3%)	43.0 (27.9%)	
M	37 (67.3%)	53 (71.7%)	12 (80.0%)	3 (100.0%)	6 (85.7%)	111.0 (72.0%)	
Keratinizing	46 (82.1%)	55 (74.3%)	11 (73.3%)	3 (100.0%)	5 (71.4%)	120 (83.3%)	0.684
Non-keratinizing	9 (17.9%)	19 (25.7%)	4 (26.7%)	0 (0.0%)	2 (28.6%)	34 (16,7%)	0.684
Nodes (TNM)							<0.001
N0	52 (92.9%)	61 (82.4%)	8 (53.3%)	1 (33.3%)	3 (42.9%)	125 (81.16%)	
N1	2 (3.6%)	2 (2.7%)	0 (0.0%)	1 (33.3%)	1 (14.3%)	6 (3.99%)	
N2	0 (0.0%)	1 (1.4%)	0 (0.0%)	0 (0.0%)	0 (0.0%)	1 (0.64%)	
N2a	0 (0.0%)	1 (1.4%)	3 (20.0%)	1 (33.3%)	0 (0.0%)	5 (3.24%)	
N2b	1 (1.8%)	7 (9.5%)	3 (20.0%)	0 (0.0%)	2 (28.6%)	13 (8.44%)	
N2c	1 (1.8%)	1 (1.4%)	0 (0.0%)	0 (0.0%)	0 (0.0%)	2 (1.29%)	
N3	0 (0.0%)	0 (0.0%)	0 (0.0%)	0 (0.0%)	0 (0.0%)	0 (0.0%)	
N3b	0 (0.0%)	1 (1.4%)	0 (0.0%)	0 (0.0%)	1 (14.3%)	2 (1.29%)	
Grading							0.251
G1	12 (20.4%)	12 (16.3%)	0 (0.0%)	0 (0.0%)	1 (14.3%)	25 (15.8%)	
G1/G2	0 (0.0%)	2 (2.7%)	0 (0.0%)	0 (0.0%)	0 (0.0%)	2 (1.3%)	
G2	42 (77.8%)	57 (77.1%)	12 (80.0%)	3 (100%)	6 (85.7%)	120 (78.5%)	
G3	1 (1.9%)	3 (4.1%)	3 (20.0%)	0 (0.0%)	0 (0.0%)	7 (4.6%)	
Neutrophils preop							0.323
Mean (SD)	5.4 (2.0)	4.8 (1.8)	5.8 (3.1)	5.1 (1.4)	5.8 (1.7)	5.2 (2.0)	
Range	1.5–10.7	1.0–10.8	2.5–15.6	3.6–6.3	4.3–9.2	1.0–15.6	
Lymphocytes preop							0.826
Mean (SD)	1.9 (0.7)	2.1 (1.5)	1.9 (0.9)	2.7 (0.2)	1.9 (0.6)	2.0 (1.2)	
Range	0.8–3.8	0.6–13.6	1.1–4.8	2.6–2.9	1.3–2.8	0.6–13.6	
Platelets preop							0.083
Mean (SD)	261.7 (76.8)	232.7 (52.2)	219.1 (82.9)	253.0 (44.0)	253.3 (72.5)	243.0 (66.9)	
Range	86.0–466.0	119.0–366.0	104.0–403.0	204.0–289.0	177.0–364.0	86.0–466.0	
SII							0.435
Mean (SD)	841.6 (635.9)	684.1 (463.9)	799.0 (626.6)	472.3 (140.8)	872.2 (546.6)	756.0 (548.4)	
Range	0.0–3132.3	123.2–2394.0	97.3–2714.4	325.7–606.6	377.2–1938.9	0.0–3132.3	
NLR							0.463
Mean (SD)	3.0 (1.6)	2.9 (1.8)	3.7 (2.5)	1.9 (0.6)	3.3 (1.5)	3.0 (1.8)	
Range	0.0–8.6	0.4–9.0	0.5–10.4	1.2–2.4	2.0–6.2	0.0–10.4	

**Table 2:** The patient values for neutrophils, lymphocytes, platelets, SII and NLR across treatment stages.

	A-Statistic	A *p*-Value	K-W Statistic	K-W *p*-Value	Mean (Preop)	Median (Preop)	Mean (Pre-1st Session)	Median (Pre-1st Session)	Mean (Post-Last Session)	Median (Post-Last Session)
Neutrophils	0.8523	0.4290	4.8896	0.0867	5.07 (1.99)	4.72 (Q1: 3.72 Q3: 5.92)	4.38 (2.13)	3.81 (Q1: 2.98 Q3: 4.79)	10.81 (41.74)	4.08 Q1: 2.88 Q3: 5.68
Lymphocytes	27.8736	1.2598	39.2799	2.9544	2.03 (0.69)	1.94 (Q1: 1.48 Q3: 2.61)	2.27 (0.69)	2.18 (Q1: 1.79 Q3: 2.91)	1.17 (0.71)	0.90 Q1: 0.64 Q3: 1.48
Platelets	1.0324	0.3593	2.7301	0.2553	237.18 (57.18)	235.00 (Q1: 186.50 Q3: 277.00)	258.78 (113.52)	251.00 (Q1: 206.50 Q3: 285.00	229.98 (99.73)	208.00 Q1: 168.0 Q3: 292.0
SII	1.5034	0.2266	12.5019	0.0019	703.93 (505.66)	563.42 (Q1: 375.48 Q3: 897.45)	645.30 (915.50)	390.55 (Q1: 289.11 Q3: 662.05)	3293.11 (13,468.30)	778.98 Q1: 409.96 Q3:1592.98
NLR	1.4366	0.2418	23.4522	8.0801	2.93 (1.85)	2.47 (Q1: 1.64 Q3: 3.69)	2.15 (1.47)	1.72 (Q1: 1.30 Q3: 2.62)	16.49 (73.65)	4.30 Q1: 2.50 Q3: 6.58

## Data Availability

The data presented in this study are available on request from the corresponding author. The data are not publicly available due to restrictions regarding the privacy of the funding protocol.
